# Supplementary material for: DMRforPairs: identifying Differentially Methylated Regions between unique samples using array based methylation profiles
Source: BMC Bioinformatics. 2014 May 15;15:141. doi: 10.1186/1471-2105-15-141 (PMC4046028; doi:10.1186/1471-2105-15-141)
Supplement: Additional file 2 — DMRforPairs output for the comparison of A431-MCF7 and NA17018-NA17105. Please start from the HTML files in each folder. Available via the BMC Bioinformatics website. [file 1471-2105-15-141-S2.zip › 1394847754114233_MOESM2_ESM/A431_MCF7/figures/33658.pdf]

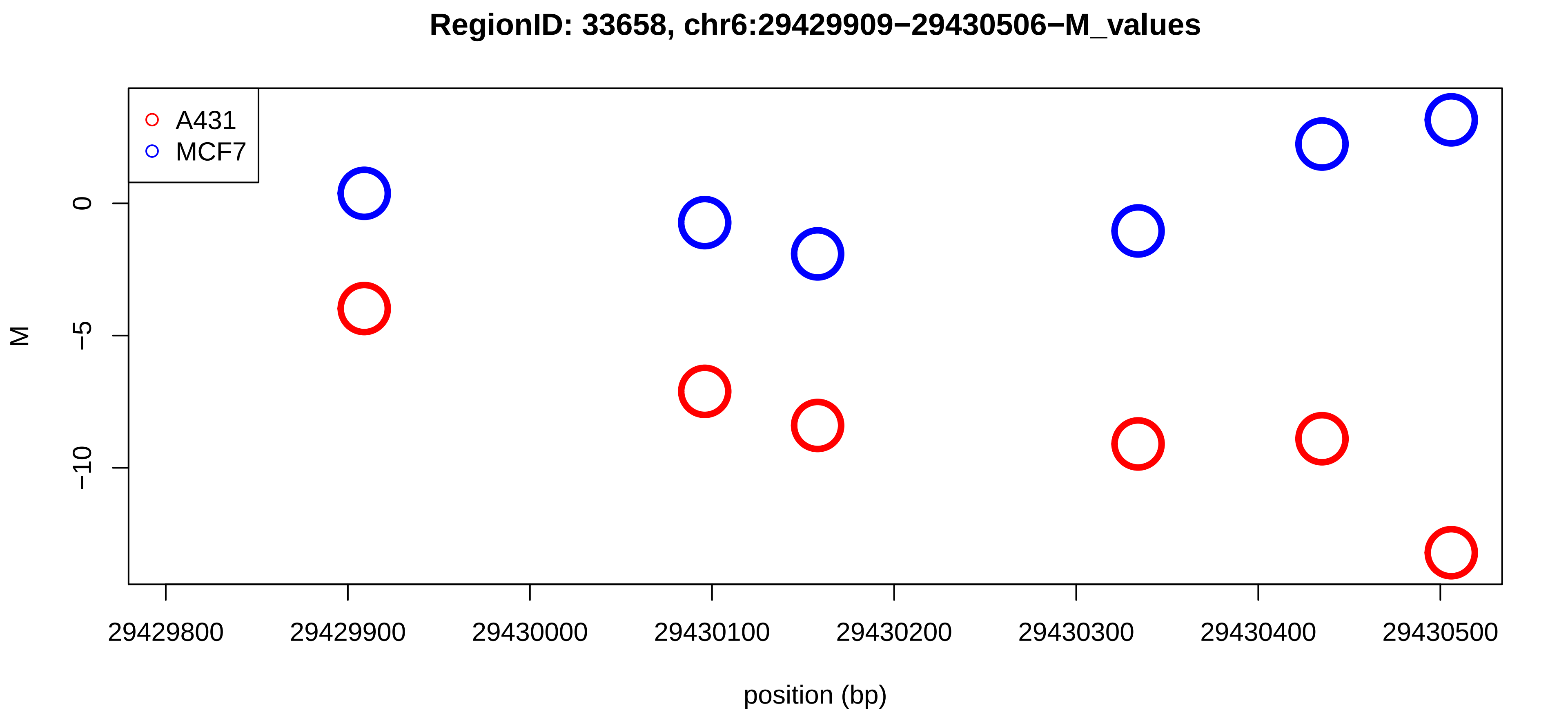
RegionID: 33658, chr6:29429909−29430506−M_values
A431
MCF7
M
0
−5
−10
29429800
29429900
29430000
29430100
29430200
29430300
29430400
29430500
position (bp)

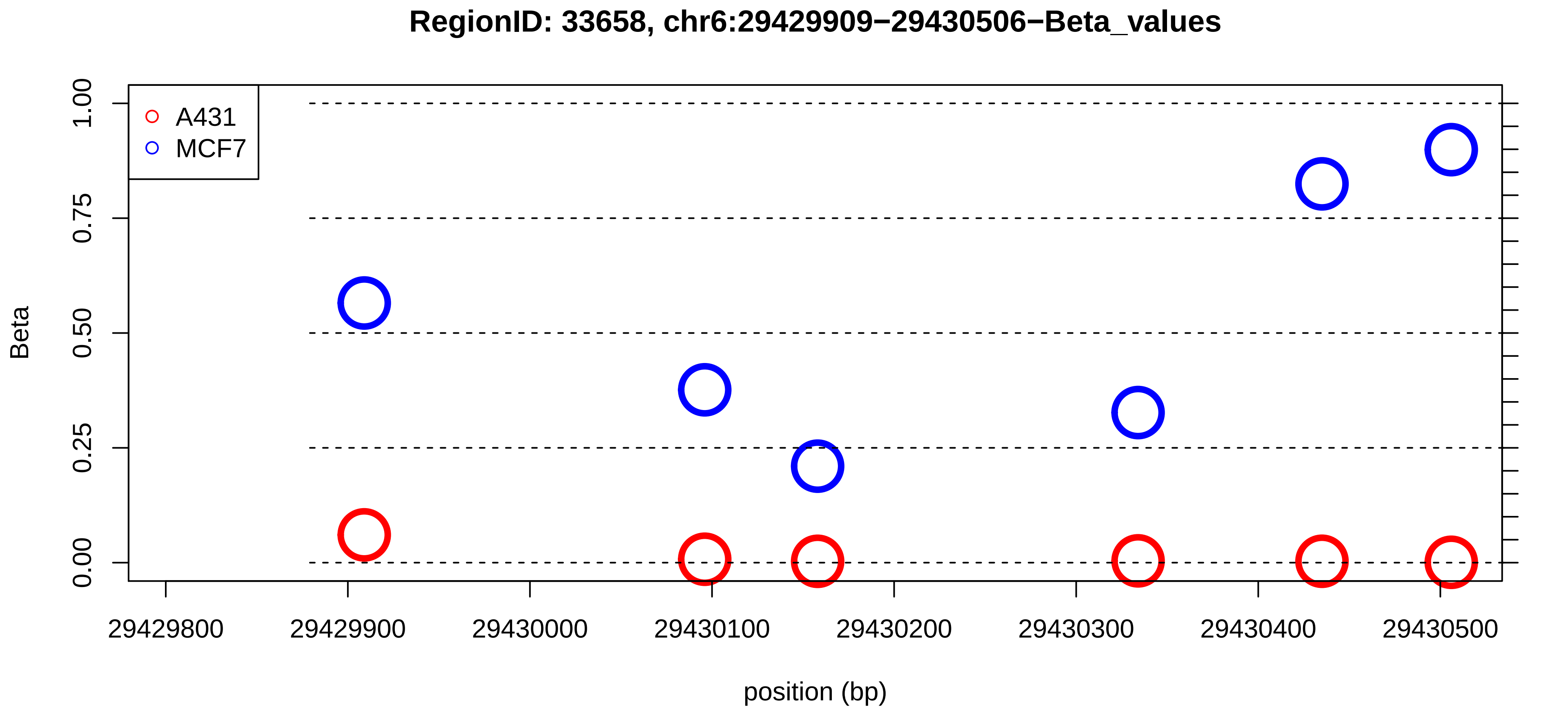
RegionID: 33658, chr6:29429909−29430506−Beta_values
A431
MCF7
Beta
0.00
0.25
0.50
0.75
1.00
29429800
29429900
29430000
29430100
29430200
29430300
29430400
29430500
position (bp)

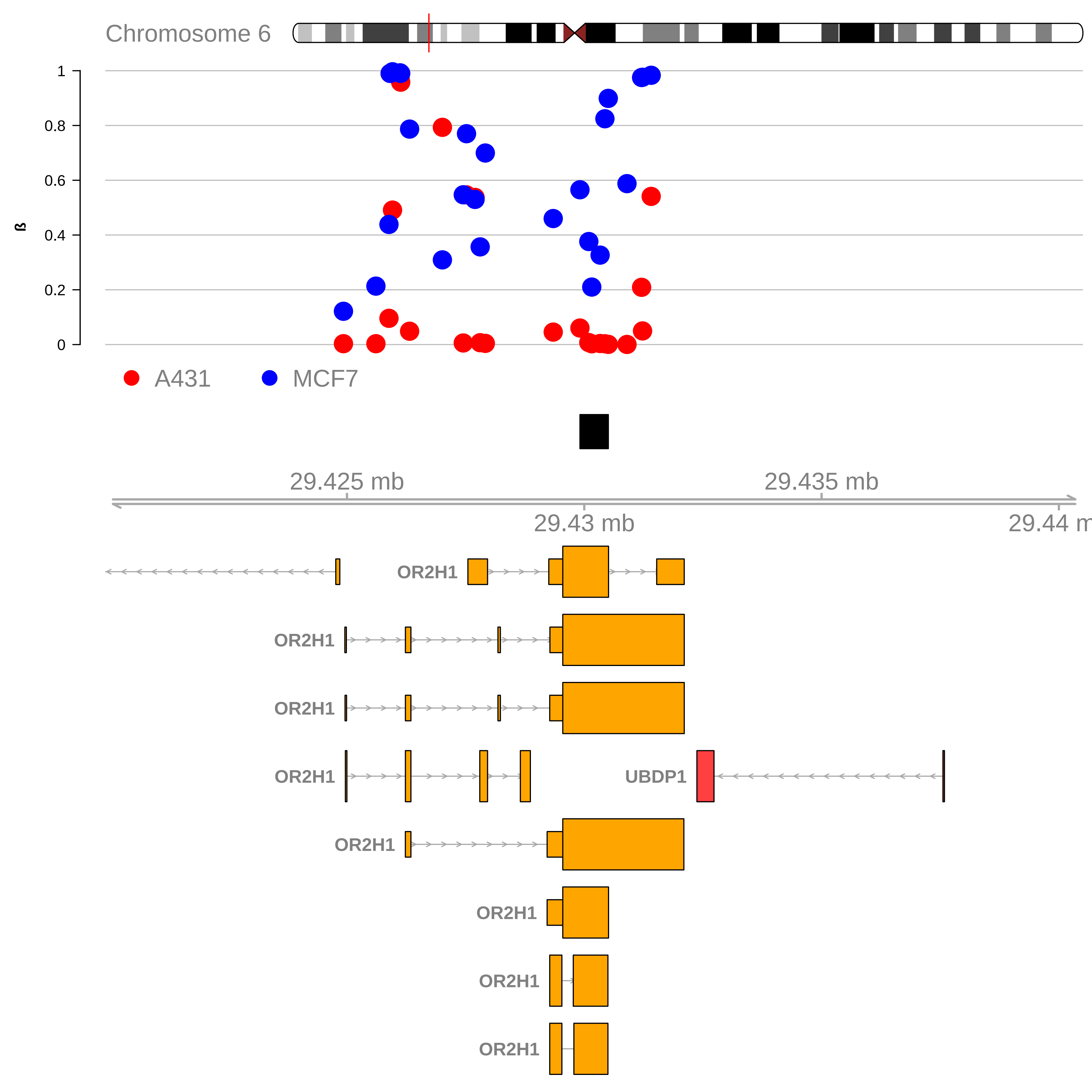

Chromosome 6
1
0.8
0.6
0.4
0.2
0
ß
A431
MCF7
29.425 mb
29.435 mb
29.43 mb
29.44 m
OR2H1
OR2H1
OR2H1
OR2H1
UBDP1
OR2H1
OR2H1
OR2H1
OR2H1
